# Movement Impairments May Not Preclude Visuomotor Adaptation After Stroke

**DOI:** 10.3390/brainsci15060619

**Published:** 2025-06-08

**Authors:** Robert Taylor Moore, Mark Andrew Piitz, Nishita Singh, Sean Peter Dukelow, Tyler Cluff

**Affiliations:** 1 Department of Clinical Neurosciences, Cumming School of Medicine, University of Calgary, Calgary, AB T2N 1N4, Canada; 2 Hotchkiss Brain Institute, University of Calgary, Calgary, AB T2N 1N4, Canada; 3 Faculty of Kinesiology, University of Calgary, Calgary, AB T2N 1N4, Canada

**Keywords:** stroke recovery, stroke rehabilitation, motor learning, motor adaptation, movement impairment, upper limb, robotics

## Abstract

Purpose: Many individuals with stroke partake in rehabilitation to improve their movements. Rehabilitation operates on the assumption that individuals with stroke can use visual feedback from their movements or visual cues from a therapist to improve their movements through practice. However, this type of visuomotor learning can be impaired after stroke. It is unclear whether and how learning impairments relate to impairments in movement. Here, we examined the relationship between learning and movement impairments after stroke. Methods: We recruited adults with first-time unilateral stroke and controls matched for overall age and sex. The participants performed a visuomotor learning task in a Kinarm exoskeleton robot. The task assessed how they adapted their reaching movements to a systematic visual disturbance that altered the relationship between the observed and actual motion of their hand. Learning was quantified as the extent to which the participants adapted their movements to the visual disturbance. A separate visually-guided reaching task was used to assess the straightness, direction, smoothness, and duration of their movements. The relationships between visuomotor adaptation and movement were analyzed using Spearman’s correlations. Control data were used to identify impairments in visuomotor adaptation and movement. The independence of these impairments was examined using Fisher’s exact tests. Results: Impairments in visuomotor adaptation (46.3%) and movement (73.2%) were common in participants with stroke (*n* = 41). We observed weak–moderate correlations between continuous measures of adaptation and movement performance (rho range: −0.44–0.58). Adaptation and movement impairments, identified using the range of performance in the control participants, were statistically independent (all *p* > 0.05). Conclusions: Movement impairments accounted for 34% of the variance in visuomotor adaptation at best. Our findings suggest that factors other than movement impairments may influence visuomotor adaptation after stroke.

## 1. Introduction

Stroke can impair upper-limb movements and create challenges in daily activities, like feeding and grooming, that jeopardize autonomy and detract from quality of life. Many individuals with movement impairments participate in rehabilitation after stroke. Stroke rehabilitation involves working with therapists through a process of guided practice to relearn arm movements that are essential for everyday tasks.

Therapists often use motor learning as a framework to promote neuroplasticity and facilitate the recovery of motor function after stroke [1,2,3]. Motor learning encompasses a range of neural and behavioral processes that improve performance through practice. These processes span from skill acquisition to motor adaptation. Skill acquisition involves learning new motor skills or sequences of movements. In contrast, motor adaptation involves modifying previously learned movements to accommodate changes in the body, environment, or demands of a task that impose systematic movement errors [4]. In the healthy nervous system, these errors engage motor learning processes that progressively adapt movements and eliminate errors to improve performance.

Stroke rehabilitation applies principles of motor learning, derived largely from healthy adults, to improve motor function through guided practice. The approach may be too simplistic. A growing body of evidence indicates that motor learning is often impaired by stroke [5,6,7,8,9,10,11,12,13,14,15,16,17]. What remains unclear is whether motor learning impairments coincide with movement impairments or have the potential to occur independently. Understanding this relationship may help to inform decisions on how to plan and deliver therapy that best reflects an individual’s motor learning and movement abilities. It may also provide a foundation for testing new interventions that aim to restore or enhance motor learning and facilitate the recovery of motor function [18,19,20].

Research on the relationship between motor learning and movement impairments has yielded mixed results. Although some studies have reported weak–moderate correlations between aspects of motor learning and traditional clinical assessments, such as the Fugl–Meyer Assessment-Upper Extremity (FMA-UE) or Chedoke–McMaster Stroke Assessment (CMSA) [5,17,21], others have not [9]. The FMA-UE and CMSA are reliable and valid assessments that use ordinal scales to score an individual’s ability to perform upper-limb movements [22,23]. They are based on visual assessment, which can make it difficult to gauge specific aspects of movement performance, like directional errors, the timing of movements, or online corrections [24]. As a result, they do not provide detailed information about movement kinematics or inform on which aspects of movement are most strongly associated with motor learning [25].

Robotic technologies can provide highly detailed, quantitative assessments of movement kinematics using spatial and temporal measures of the straightness, initial direction, smoothness, and timing of movements [24,26]. These devices can also help to identify impairments in the ability to learn and perform movements based on the range of behavior in control participants matched for demographic variables, such as age and/or sex [25,27,28,29,30].

Here, we investigated the relationship between a specific type of motor learning known as visuomotor adaptation and visually-guided reaching assessed with a robotic reaching task. Visuomotor adaptation refers to the process of reducing movement errors that are evoked by a systematic visual disturbance. This type of learning is thought to capture some of the challenges that individuals with stroke encounter in their daily lives, like adjusting to new eyeglasses, grooming in a mirror, or using a trackpad on a laptop computer [3,17]. We also assessed movement performance in the upper limb with standardized clinical scales (FMA-UE and CMSA). The objective was to determine how performance and impairments in a visuomotor adaptation task were related to robotic assessments of visually-guided reaching and clinical measures of movement performance.

## 2. Materials and Methods

### 2.1. Participants

Participants with stroke were recruited from inpatient rehabilitation units at Foothills Medical Centre and Carewest Dr. Vernon Fanning Centre in Calgary, AB, Canada. We also recruited participants with stroke who consented to being contacted after discharge from the inpatient rehabilitation units. The inclusion criteria were 18 years of age or older with a diagnosis of first-time ischemic or hemorrhagic stroke. The exclusion criteria were stroke in the cerebellum, a history of other neurological conditions (e.g., cerebellar ataxia, multiple sclerosis, Parkinson’s disease), upper-limb musculoskeletal injuries that could impede on the ability to perform the experimental tasks, difficulty comprehending and/or following task instructions, or the presence of motor apraxia [31]. We also recruited control participants from the University of Calgary and surrounding communities. Controls were considered eligible if they had no history of neurological conditions or musculoskeletal injuries in the upper limb that could impede on their ability to perform the experimental tasks. The stroke and control samples were matched for overall age and sex.

### 2.2. Ethics Approval and Consent to Participate and Publish

The study protocol was approved by the Conjoint Health Research Ethics Board at the University of Calgary. All methods were performed in accordance with relevant guidelines and regulations. The participants provided written informed consent before they performed the tasks. Consent to publish data was obtained from all participants.

### 2.3. Robotic Apparatus

The participants performed planar reaching movements with their arm supported against gravity by a robotic exoskeleton (Kinarm™, Kingston, ON, Canada). The participants with stroke performed the tasks with their more affected arm, as this is the arm that typically undergoes rehabilitation. The controls performed the task with their dominant arm, as studies have revealed similar patterns of adaptation across both arms in healthy adults [32,33,34,35,36]. The robot recorded shoulder and elbow joint motion as the participants moved in a near-frictionless environment. The robot was paired with a visual display that allowed for the participants to interact with virtual targets displayed in their workspace. A hand-feedback cursor (0.8 cm diameter) was also displayed in the participant’s workspace and aligned to the tip of their index finger unless specified otherwise. Direct vision of the arm and hand was blocked by a metal shutter and cloth bib throughout the experiment. We assessed adaptation using a visuomotor rotation task (VMR) [17] and reaching using a visually-guided reaching (VGR) task [25].

### 2.4. Visuomotor Rotation (VMR) Task

**Figure 1:**
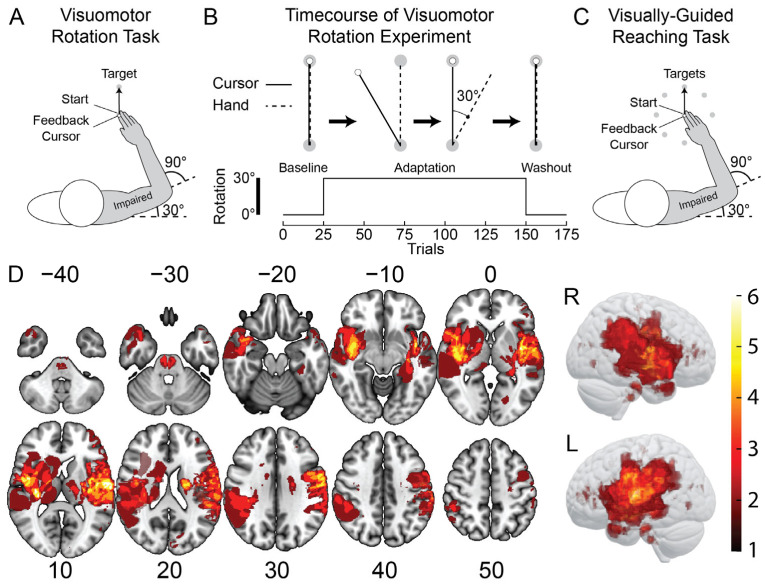
General task design and lesion characteristics. (**A**) Participants performed reaching movements from a start position to a single target while adapting to a visuomotor rotation (VMR). Initial arm configuration at the start position was the same as the VGR task described below. (**B**) Participants began the VMR task with 25 reaching trials with a small cursor displayed over the position of their occluded fingertip (veridical feedback). In the adaptation phase, we applied a 30° counter-clockwise rotation of the feedback cursor relative to the center or the start position. This meant that, as participants reached straight, the cursor travelled 30° leftward. Participants adapted their reaching direction to the rotation over 125 trials. Finally, participants performed 25 reaches with veridical feedback to washout the effects of adaptation. (**C**) Participants also performed a center-out, visually-guided reaching (VGR) task. Eight targets were spaced radially 10 cm from a central start position. The central start position was located in front of the participant so that their initial arm configuration was 30° of shoulder flexion and 90° of elbow flexion relative to the upper arm. (**D**) Lesion overlap maps of all participants with stroke (neurological display convention; *n* = 40). MNI coordinates are labeled and displayed below each slice. The color bar indicates the number of participants with damage in each voxel (brighter colors indicate regions in which more participants had stroke-related damage).

Visuomotor adaptation was assessed in the upper limb using a VMR task [17]. The general design of the task is shown in Figure 1A. The participants began each trial by guiding their hand-feedback cursor into a start position (2 cm diameter). The participants held the start position for a random amount of time (750 ± 500 ms, uniformly distributed). A single target then appeared 10 cm directly in front of the start position. The participants were instructed to make smooth and accurate movements from the start position to the target. The participants were then required to stabilize their cursor in the target for 1000 ms. The start position reappeared after the stabilization period, cueing the participant to return to begin the next trial. The trials were self-paced, and reaction times were not constrained.

The VMR task consisted of baseline, adaptation, and washout phases (Figure 1B). The participants performed 25 movements in the baseline phase to quantify their typical reaching movements in the robot. The feedback cursor was aligned to the tip of the participant’s index finger. Throughout the adaptation phase, the position of the feedback cursor was rotated 30° counter-clockwise relative to the center of the start position (Figure 1B). The rotation caused the cursor to move 30° leftward of a straight line joining the start position and target when moving directly forward to the target. The participants performed 125 reaching movements with the rotated hand-feedback cursor in the adaptation phase. The cursor was then abruptly realigned with their index fingertip, and the participants performed 25 reaching movements to washout the effects of adaptation.

Adaptation was quantified by measuring the signed initial reach direction (*IRD*) of each movement. The position of the hand was recorded 150 ms after movement onset and used to calculate the angular deviation from a straight line joining the start position and target. This approach allowed for us to examine each participant’s planned movements while reducing the influence of corrective movements that take place throughout the reach [37,38]. *IRDs* were baseline reduced for each participant after verifying there were no differences in the average direction of baseline movements between the control sample and participants with stroke. The *IRD* is a sensitive marker of motor adaptation [39,40] and relevant for detecting impairments after stroke [16,17]. While other metrics (e.g., hand path length, movement time) may offer additional insights into the process of adaptation, the task required the participants to complete the reach by moving their cursor into the target. Thus, lower amounts of adaptation (lower initial reach direction) will require larger online corrections, resulting in longer hand path lengths and movement times. Our selective approach, focused on *IRD*, enabled the assessment of adaptation while minimizing the risk of Type I errors or punitive corrections for multiple comparisons.

Performance in the VMR task was quantified using the degree of adaptation achieved during *Initial* and *Final Adaptation* [17]. We also quantified the number of trials required for the participants to adapt to normative levels (*Trials to Adapt*). Impaired visuomotor adaptation was operationalized as displaying impairment on at least one of the following measures of adaptation.

*1. Initial Adaptation:* We quantified *Initial Adaptation* as the average *IRD* during the first 15 adaptation trials. This measure quantifies how much the participants adapted their movements when first exposed to the rotated visual feedback.

*2. Final Adaptation:* We quantified *Final Adaptation* as the average *IRD* during the last 15 adaptation trials. This measure quantifies the extent to which the participants adapted their movements to counter the rotated visual feedback after 110 practice trials.

*3. Trials to Adapt:* We calculated the normative range containing 95% of the control sample for *Final Adaptation* (one-tailed). The lower bound (i.e., 5th percentile) of this range was used to quantify the number of trials that each participant required to adapt to the rotation for 15 consecutive trials. The first trial to meet this criterion was taken as the number of *Trials to Adapt*. This measure assesses how quickly individuals adapted and performed consistently above the lower bound of normal levels. The participants who did not adapt to within the normal range were assigned a value of 125 trials, corresponding to the length of the adaptation phase [17].

The VMR task is not a standard Kinarm task, unlike the VGR task detailed below, and does not have an existing normative dataset. We started developing a normative dataset (*n* = 41) for the VMR task, matched for overall age and sex, and calculated the normative ranges for each measure of adaptation based on the performance of 95% of the control sample. For *Initial* and *Final Adaptation*, impairment was identified using the lower bound (i.e., 5th percentile) as a threshold for identifying individuals who adapted less than 95% of the control sample. The upper bound (i.e., 95th percentile) of *Trials to Adapt* was used to identify individuals who required more *Trials to Adapt* than 95% of the control sample. Individuals who required more *Trials to Adapt* were considered impaired on this measure.

### 2.5. Visually-Guided Reaching (VGR) Task

Reaching was assessed using the VGR task, which has been shown to be a valid and reliable assessment of motor impairments in adult stroke survivors [25]. The design of the task is shown in Figure 1C. The participants began each trial by holding their hand-feedback cursor in a start position (2 cm diameter) for a random amount of time (1500 ± 250 ms, uniformly distributed). The start position was the same as the VMR task. One of eight targets (2 cm diameter) then appeared 10 cm away. The participants were instructed to reach to the target as quickly and accurately as possible. After maintaining the target for the same brief hold period (1500 ± 250 ms, uniformly distributed), the start position reappeared on the screen, and the participants returned to begin the next trial. Each target was presented once per block. The targets were presented in pseudorandom order, such that the same target was never encountered in consecutive trials. The participants performed eight movements to each target for a total of 64 trials.

A host of measures can be derived from the VGR task to assess visually-guided reaching (see KST summary, www.kinarm.com). The measures have been shown to be sensitive to impairments after stroke [25,27,41]. However, a recent consensus statement recommended examining fewer measures to avoid issues with multiple comparisons that may confound the interpretation of findings [24]. Therefore, we selected four measures to characterize visually-guided reaching. The specific measures, outlined below, were selected based on guidelines put forward by the International Stroke Recovery and Rehabilitation Roundtable (SRRR) [24].

*1. Path Length Ratio (PLR):* The *Path Length Ratio* (*PLR*) examines the straightness of movement on each trial. *PLR* was defined as the ratio between the distance travelled by the hand and distance between the start position and target [24,25]. Perfectly straight movements receive a ratio of one, whereas values greater than one reflect movements with larger curvature.

*2. Initial Direction Error (IDE):* Participants with stroke often make directional errors when they initiate reaching movements [25]. *Initial Direction Errors* (*IDE*) were quantified as the unsigned angular deviation of the hand path relative to a straight line between the start position and target on each trial. The *IDE* was quantified at the completion of the initial movement, which was defined as the time of the first local speed minimum after the first speed maximum [25]. Larger values reflect greater directional errors at the end of the initial movement.

*3. Speed Maxima Count (SMC):* Reaching movements are often segmented after stroke and comprise more sub-movements than reaching movements performed by healthy adults [42]. We measured the *Speed Maxima Count* (*SMC*) as the number of speed peaks that occurred during each movement [25]. This measure provides information about the smoothness of movement by assessing the number of online corrections as participants reach to targets in their workspace [24,43]. Smaller values reflect smoother movements with fewer corrections.

*4. Movement Time (MT):* Previous studies have shown that *Movement Time* is often elevated after stroke and can be used as a general measure of visually-guided reaching [25,41,42]. *Movement Time* was defined as the time between the onset and offset of each movement. Movement onset was defined as the first time point after the cursor left the start position and (1) the participant’s hand speed exceeded the 95th percentile of their hand speed when stabilized in the start position on that trial, or if this criterion was not met, (2) the participant’s hand speed exceeded the 50th percentile of the hand speed when holding the start position across all movements performed in the VGR task [25]. Movement offset was defined as the time when the cursor entered the target and hand speed was lower than criterion 1 or 2 [25].

The SRRR has also recommended including a summary measure of overall visually-guided reaching performance [24]. Therefore, we quantified a *VGR Task Score* based on 11 measures from the VGR task (Supplementary Materials S1).

*5. VGR Task Score:* The VGR task is a standard Kinarm task with an existing normative dataset. Each participant’s *VGR Task Score* was calculated using a normative dataset that accounts for age, sex, and handedness (613 exams from 307 controls, 155 female, age = [19–84]). The procedure is automated by the Dexterit-E 3.9 software (Kinarm™, Kingston, ON, Canada). We provide a summary here (further details available in Kinarm Standard Task (KST) summary, www.kinarm.com). First, each task measure was converted to a z-score based on the distribution of normative data for that parameter. The z-scores were converted to zeta-scores, such that the best zeta-score on any individual measure was zero and larger values reflect poorer performance [44]. The zeta-scores were then used to calculate the root–sum–square (RSS) across all of the individual task measures [45]. Finally, the RSS distances were renormalized to z-scores using Box–Cox transformations and once again converted to zeta-scores [44,45]. A *VGR Task Score* greater than 1.96 was outside of the normative range (95% of the control data < 1.96) and considered as an impairment in visually-guided reaching.

### 2.6. Imaging and Lesion Delineation

Clinical MRI (*n* = 35) scans were obtained a median of 1 day (range [0–36]) post-stroke as a part of acute stroke imaging protocols at the Foothills Medical Centre [46,47]. MRI included T2 fluid-attenuated inversion recovery (FLAIR), diffusion weighted imaging (DWI), and apparent diffusion coefficient (ADC) sequences. Susceptibility weighted imaging (SWI) or gradient echo (GRE) sequences were also obtained for the participants with hemorrhagic stroke. An MRI was not performed if there was a well-defined infarct on the clinical CT. In such cases (*n* = 5), lesion characteristics were quantified from the CT scan (median days post-stroke = 1, range [1–9] days) [46,47,48]. The procedure is consistent with standard acute stroke imaging protocols at the Foothills Medical Centre [46,47]. Imaging was not available for one participant.

A trained assessor delineated the stroke lesions on the T2-FLAIR or non-contrast CT using MRIcron software (https://www.nitrc.org/projects/mricron; accessed: January 2022) [49]. DWI and ADC scans were used to identify ischemic brain lesions. SWI or GRE sequences were used to characterize brain regions damaged by intracranial hemorrhage [46]. This procedure produced a volume of interest (VOI) encompassing the region of the brain impacted by stroke. VOIs were verified by a stroke neurologist blinded to the purpose and results of this study. Next, the clinical toolbox [50] in SPM12 [51] was used to register all VOIs to the spm152 anatomical template in MRIcroGL 14.1 (https://www.mccauslandcenter.sc.edu/mricrogl; accessed: January 2022) [49]. Distortion and warping of damaged tissue during the registration process were prevented by applying a cost function mask over the areas impacted by stroke [52]. The registered VOIs were inspected and compared to the original image to ensure accuracy. VOIs were then used to create an overlap map of the lesions present in the sample of participants with stroke.

### 2.7. Clinical Assessments

Clinical assessments were performed by a research therapist a median of 3 days (range [0–11]) from when the participants with stroke performed the robotic assessment. The therapist was blinded to the purpose and results of this study. The therapist performed the following assessments: Fugl–Meyer Assessment of Motor Recovery—Upper Extremity Motor Assessment (FMA-UE, assesses motor function of the arm and hand) [53], Chedoke–McMaster Stroke Assessment—Arm Impairment Inventory (CMSA, measures physical impairment in the arm) [22], Purdue Pegboard Test (PPT, assesses finger dexterity and gross movement of the arm, hand, and fingers; LaFayette Instrument Co., LaFayette, IN, USA) [54], Medical Research Council—strength score composite (MRC, measure of arm strength) [55], Modified Ashworth Scale (MAS, measures spasticity of the elbow flexors) [56], Thumb Localization Test (TLT, assesses proprioceptive impairments in the arm) [57], and Functional Independence Measure (FIM, measures independence in performing activities of daily living) [58]. The FMA-UE and CMSA were used to examine the relationship between visuomotor adaptation and traditional clinical measures of movement performance. All other assessments (PPT, MRC, MAS, TLT, and FIM) were included to characterize the sample of participants with stroke.

### 2.8. Statistical Analysis

Age was compared across the stroke and control samples using bootstrap hypothesis tests (two-tailed). Sex was compared across the stroke and control samples using chi-squared tests. Measures of adaptation were compared across the stroke and control samples using two-sample bootstrap hypothesis tests (one-tailed) [59]. Note that all bootstrap tests were performed by resampling the data 99,999 times in agreement with recommendations for hypothesis testing [60]. Previous work has shown that reduced adaptation is associated with lower scores on clinical assessments of movement performance (i.e., FMA and CMSA) [17]. We performed Spearman’s correlations (one-tailed) to test how measures of adaptation were related to individual measures and overall *Task Score* in visually-guided reaching, FMA-UE scores, and CMSA scores. We bootstrapped the correlation analysis by resampling 99,999 times with replacement to obtain confidence bounds on Spearman’s rho (effect size). The correlations were interpreted based on statistical significance (*p*-value) and the effect size (rho) using established guidelines [61,62]. Exact effect sizes (rho), bootstrapped confidence intervals on effect sizes, and *p*-values are reported in the corresponding figures.

Normative data were used to identify individuals with impairments in adaptation, reaching, both, or neither task. Fisher’s exact tests of independence were then used to examine the categorical relationship (i.e., statistical independence) between impairments identified in visuomotor adaptation and reaching. Fisher’s exact test assesses whether there is a non-random association between categorical variables in a 2 × 2 contingency table. In our tasks, there were four possible outcomes: impaired visually-guided reaching, impaired in measures of visuomotor adaptation, impaired in both, or unimpaired in both. This analysis was repeated to examine the categorical relationship between impairments in adaptation and movement impairments assessed on the FMA (impaired: FMA score < 66) and CMSA (impaired: CMSA score < 7). Odds ratios, confidence intervals, and *p*-values are reported in the corresponding figures. In certain cases, one of the counts in the 2 × 2 contingency table for the Fisher’s exact test was zero. In these instances, we repeated the analyses with Barnard’s test (Supplementary Materials S2) based on recommendations [63]. Bonferroni–Holm methods were applied to control for the family-wise error rate arising from multiple statistical comparisons and correlations [64]. We present the corrected *p*-values throughout the text. The threshold for significance was set to α = 0.05. All analyses were performed using custom scripts developed in MATLAB 2021b (MathWorks, Natick, MA, USA).

## 3. Results

A total of 82 participants were included in this study (41 individuals with stroke and 41 controls). Figure 1D shows the lesion characteristics of the stroke sample. Demographic information and scores from the clinical assessments are displayed in Table 1. Age (bootstrap: difference = −4.37, CI [−9.24, 0.634], *p* = 0.0918) and sex (chi-squared: difference = 12.2%, CI [−9.08%, 32.1%], X^2^(1) = 1.57, *p* = 0.210) did not differ significantly across the participants with stroke and controls (Table 1). The median time between stroke and robotic assessment was 31 days (range [3, −1102] days), placing most of our sample within the subacute phase of stroke recovery (90.2%) and participating in inpatient therapy when they performed the experimental tasks [65].

### 3.1. Representative Participants

**Figure 2:**
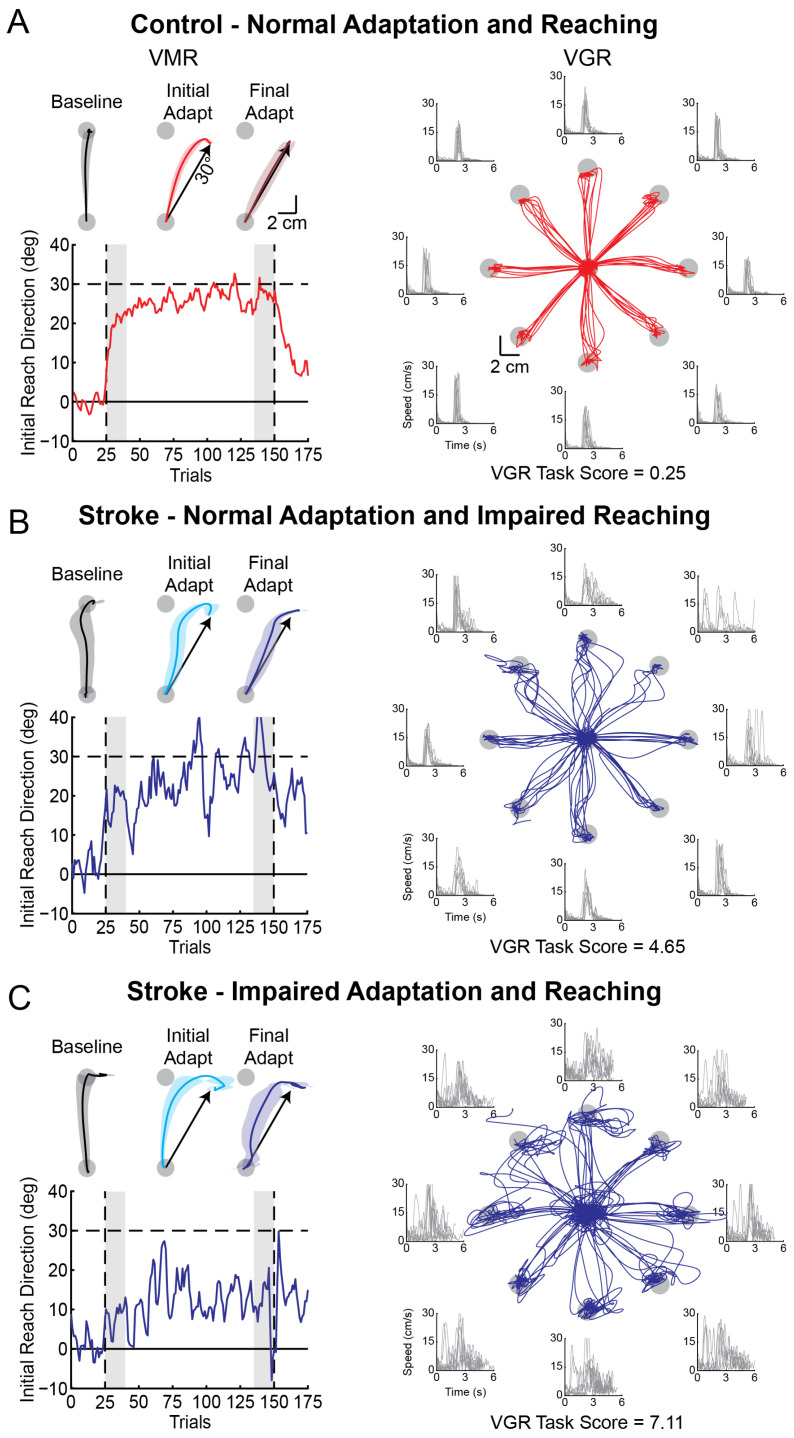
Exemplar hand paths in the VMR and VGR tasks with adaptation curves for a representative control (red), a participant with stroke who had reaching impairments (blue), and a participant with stroke who had impaired adaptation and reaching movements (blue). (**A**) **Left:** the average hand paths during *Baseline*, *Initial Adaptation*, and *Final Adaptation* (shaded regions = SD) are shown for the VMR task. **Left:** adaptation curve describing the trial-by-trial change in initial reach direction throughout the VMR task is shown for the control. Shaded regions represent the trials in which *Initial* and *Final Adaptation* were calculated. **Right:** hand paths and hand speed profiles are shown for a control who performed well on the VGR task. (**B**) Exemplar participant with stroke who displayed normal adaptation and impaired reaching. (**C**) Exemplar participant with stroke who displayed impaired adaptation and reaching. Data in (**B**,**C**) are presented in the same format as (**A**).

Figure 2 shows hand paths and adaptation curves from the VMR task as well as hand paths and speed profiles from the VGR task. Data are shown for a representative control and two participants with stroke. Figure 2A shows data from a representative control participant in the visuomotor rotation (VMR) and visually-guided reaching (VGR) tasks. The representative control made relatively straight movements from the start position to the target in the baseline phase of the VMR task. They began adapting to the visuomotor rotation by altering the initial direction of their reaching movements rightward (i.e., clockwise) in *Initial Adaptation*. In *Final Adaptation*, the control participant displayed near complete adaptation by reaching ~30° clockwise (rightward of target) to counter the visuomotor rotation. The control participant displayed rapid, direct movements in the VGR task, with few corrective sub-movements denoted by relatively few peaks in their hand speed profile (Figure 2A). Figure 2B shows a representative participant with stroke with normal adaptation in the VMR task and impaired reaching in the VGR task (*VGR Task Score* > 1.96). In the VMR task, this participant adapted well to the rotated visual feedback in *Initial* and *Final Adaptation*. In the VGR task, the participant made initial direction errors and, as a result, performed several corrective sub-movements denoted by numerous peaks in their hand speed profiles. The other representative participant with stroke demonstrated impairments in adaptation and reaching (Figure 2C). This participant displayed impairments in *Initial* and *Final Adaptation* in the VMR task. They adapted their *IRD* very little when they encountered the rotation and relied on online corrections to move their cursor into the target. This resulted in curved hand paths, with corrective movements becoming evident as pronounced rightward displacement of their hand path to move their cursor into the target. These curved hand paths were still present in *Final Adaptation*. In the VGR task, the participant displayed large initial direction errors and curved reaching paths, with numerous corrective sub-movements visible as distinct peaks in their hand speed profile.

### 3.2. Group-Level Performance in Visuomotor Adaptation and Visually-Guided Reaching Tasks

**Figure 3:**
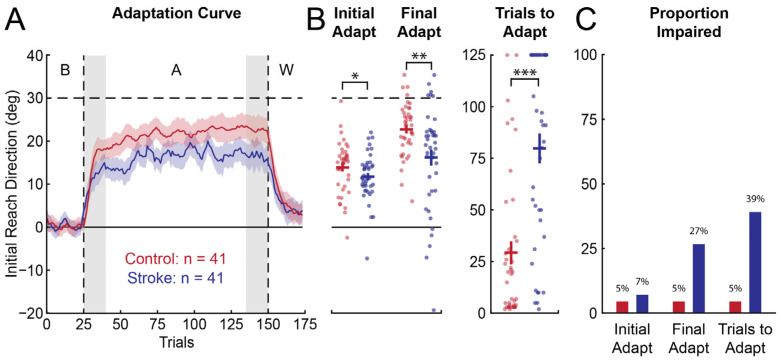
Adaptation curves and proportion of participants that were impaired on each parameter in the VMR task. (**A**) Averaged adaptation curves for controls (red) and participants with stroke (blue). Adaptation data were smoothed using a moving average filter (window length = 5; overlap = 4). Line represents the mean, and shaded region is the SE. Gray regions indicate when *Initial Adaptation* and *Final Adaptation* were averaged. (**B**) Mean (horizontal bar), SE (vertical bar), and individual data for *Initial Adaptation*, *Final Adaptation*, and *Trials to Adapt*. Bootstrap hypothesis tests were performed to test for differences in measures of adaptation. (**C**) The proportion of participants that were impaired on each parameter of adaptation. Chi-square tests were performed to test for differences in proportions. * indicates *p* < 0.05. ** indicates *p* < 0.01. *** indicates *p* < 0.001.

The average *IRD* in the baseline phase of the VMR task did not differ significantly between the participants with stroke and controls (bootstrap: difference = −0.304°, CI [−1.12°, 0.641°], *p* = 0.290; Figure 3A). However, the participants with stroke displayed more variable *IRD* (standard deviation; bootstrap: difference = 3.04°, CI [1.46°, 4.64°], *p* < 0.001). The participants with stroke adapted less than the controls, on average, both in *Initial Adaptation* (bootstrap: difference = −2.19°, CI [−4.64°, −0.247°], *p* = 0.0436; Figure 3A,B) and *Final Adaptation* (bootstrap: difference = −6.48°, CI [−10.6°, −2.51°], *p* = 0.00155; Figure 3A,B). They also required significantly more *Trials to Adapt* than the controls (bootstrap: difference = 50.4, CI [32.5, 67.5], *p* < 0.001; Figure 3B). Within the sample of participants with stroke, 7.32% had impairments in *Initial Adaptation*, 26.8% had impairments in *Final Adaptation*, and 39.0% had impairments in *Trials to Adapt* (Figure 3C). Nearly half of the participants with stroke had impairments in at least one measure of adaptation (46.3%).

Performance in the VGR task was variable amongst the participants with stroke (*VGR Task Score*: mean = 3.56, range = [0.210, −7.11]). Consistent with previous reports, over 70% of the participants with stroke had impairments in reaching (73.2%; *VGR Task Score* > 1.96). Within our sample, 56.1% of the participants with stroke had impairments on *PLR*, 65.9% had impairments on *IDE*, 46.3% had impairments on *SMC*, and 51.2% had impairments on *MT*.

### 3.3. Visuomotor Adaptation Correlates with Some Measures of Visually-Guided Reaching

**Figure 4:**
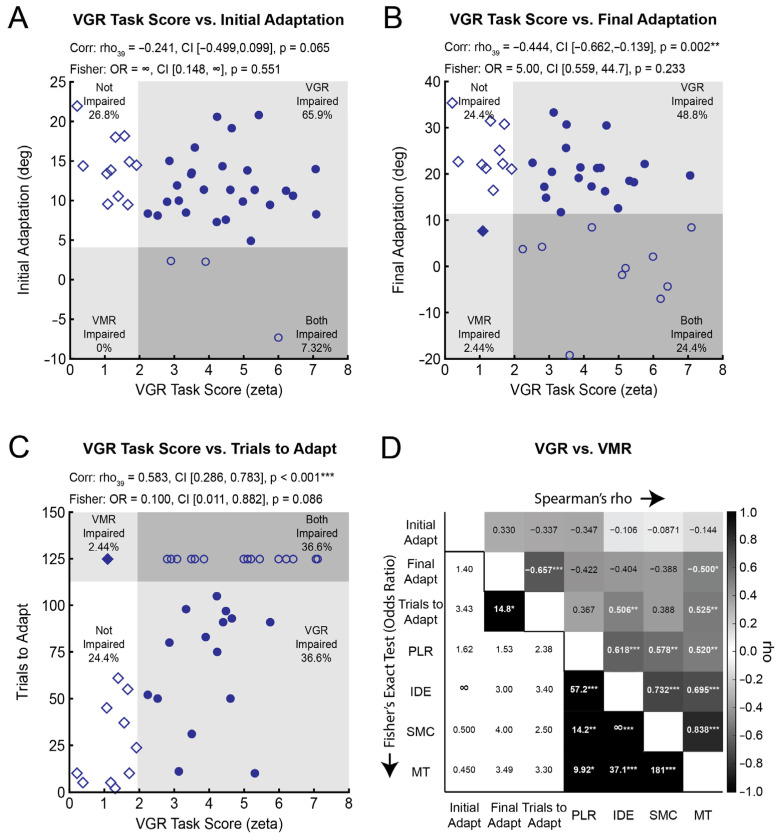
Scatterplots of *VGR Task Score* and (**A**) *Initial Adaptation*, (**B**) *Final Adaptation*, and (**C**) *Trials to Adapt* for participants with stroke. Scatterplots have been divided into 4 quadrants: participants with normal reaching and adaptation (open diamond), participants with impaired adaptation (solid diamond), participants with impaired reaching (solid circle), and participants with impaired reaching and adaptation (open circle). Spearman’s correlations indicate the strength of ranked associations. Fisher’s exact tests assessed for categorical relationships between impairments. (**D**) Spearman’s correlations and Fisher’s exact tests of independence for individual measures derived from the VGR and VMR tasks. Upper right: Spearman’s rho values are presented, and darker boxes indicate stronger correlations. Bold white numbers indicate significant correlations between variables (*p* < 0.05 after Bonferroni–Holm corrections—21 correlations). Bottom left: Odds ratios from the Fisher’s exact tests of independence are presented, and black boxes with bold white numbers indicate significant categorical associations between variables (*p* < 0.05 after Bonferroni–Holm corrections—21 tests). * indicates *p* < 0.05. ** indicates *p* < 0.01. *** indicates *p* < 0.001.

Next, we determined if individual measures of adaptation correlated with the *VGR Task Score*—an overall measure of performance in visually-guided reaching. The associations were generally of weak-to-moderate strength. *Initial Adaptation* did not correlate significantly with *VGR Task Score* (Figure 4A). We observed a significant negative correlation between *Final Adaptation* and *VGR Task Score*, demonstrating that better visually-guided reaching (i.e., lower *Task Score*) was associated with higher levels of *Final Adaptation* (Figure 4B). A significant positive relationship was observed between *Trials to Adapt* and *VGR Task Score* (Figure 4C), such that better performance in the VGR task (i.e., lower *Task Score*) was associated with fewer *Trials to Adapt*. Few correlations between individual measures of adaptation and visually-guided reaching (*PLR*, *IDE*, *SMC*, and *MT*) reached statistical significance (rho range: −0.500–0.525; Figure 4D). More *Trials to Adapt* was moderately associated with greater *IDE*. Less *Final Adaptation* and more *Trials to Adapt* were moderately associated with longer *MT*.

### 3.4. The Independence of Impairments in Visuomotor Adaptation and Reaching

Figure 4A–C show the proportion of participants with impairments in measures of adaptation, *VGR Task Score*, both, or neither. Impairments in *VGR Task Score* were independent from impairments in *Initial Adaptation*, *Final Adaptation*, and *Trials to Adapt*. Impairments in adaptation were also independent from impairments on individual measures of reaching kinematics (*PLR*, *IDE*, *SMC*, and *MT*) derived from the VGR task (Figure 4D; Supplementary Materials S2).

### 3.5. The Relationship Between Visuomotor Adaptation, FMA Scores, and CMSA Scores

**Figure 5:**
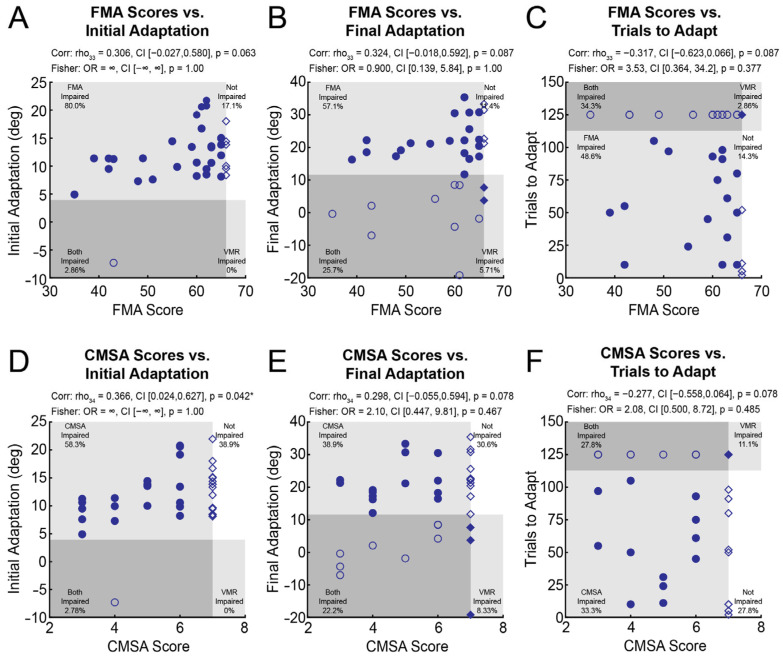
Scatterplots of Fugl–Meyer Assessment (FMA) scores and (**A**) *Initial Adaptation*, (**B**) *Final Adaptation*, and (**C**) *Trials to Adapt* for participants with stroke. Each panel is divided into 4 quadrants: participants with normal FMA scores (FMA = 66) and adaptation (open diamond), participants with impaired adaptation (solid diamond), participants with impairments on the FMA (FMA < 66; solid circle), and participants with impairments on the FMA and adaptation (open circle). Scatterplots of Chedoke–McMaster Stroke Assessment (CMSA) scores and (**D**) *Initial Adaptation*, (**E**) *Final Adaptation*, and (**F**) *Trials to Adapt* are also presented for participants with stroke. Data in (**D**), (**E**), and (**F**) are presented in the same manner as (**A**), (**B**), and (**C**), respectively. Spearman’s correlations indicate the strength of ranked associations. Fisher’s exact tests were included to test for categorical relationships. * indicates *p* < 0.05.

We also examined how measures of adaptation correlated with traditional measures of motor function in the upper extremity (i.e., FMA-UE and CMSA scores). The relationships between measures of adaptation and FMA-UE scores were non-significant (Figure 5A–C). We did, however, observe a significant positive correlation between *Initial Adaptation* and CMSA scores (Figure 5D), such that better scores on the CMSA were associated with greater *Initial Adaptation*. Correlations between *Final Adaptation* and CMSA scores as well as *Trials to Adapt* and CMSA scores were non-significant (Figure 5E,F). Impairments in adaptation were statistically independent from movement impairments assessed by the FMA and CMSA (Figure 5).

Supplementary analyses were performed to rule out the possibility that the results were influenced by differences in task design (single target VMR, multitarget VGR), definition of initial direction errors, or side of the more affected limb. We repeated the analysis using data from the baseline phase of the VMR task to test whether different task designs and methods for calculating these variables influenced our results (Supplementary Materials S3). Specifically, we re-computed *IDE* (unsigned angular deviation) in the baseline phase of the VMR task at 150 ms after movement onset (used for *IRD* in the VMR task) and the original VGR method (unsigned angular deviation based on the first local speed minimum following the first speed peak). This analysis qualitatively reproduced the main findings. Performance in the baseline phase of the VMR task was only weakly to moderately correlated with performance in the adaptation phase of the same task, and impairments, quantified relative to the range of performance in control participants, were statistically independent (Supplementary Materials S3). The results held when controlling for the side of the more affected arm (dominant vs. non-dominant; Supplementary Materials S3). We also examined the relationship between adaptation and baseline movement variability in the VMR task. The results showed a moderate, positive association between *Trials to Adapt* and movement variability, such that the participants with more variable baseline movements required more *Trials to Adapt* to the visuomotor rotation (Supplementary Materials S3). Previous studies have been conducted exclusively in right-handed participants [11,12,13,15]. Repeating the analyses with only the right-handed participants with stroke (*n* = 39) replicated the main findings, highlighting that our sample of left-handed participants (*n* = 2) did not appreciably change the results (Supplementary Materials S3).

## 4. Discussion

We examined how a specific type of motor learning known as visuomotor adaptation (VMR) relates with robotic measures of visually-guided reaching (VGR) and traditional clinical assessments of movement performance (FMA and CMSA) in participants with stroke. The results revealed weak-to-moderate associations between individual measures of adaptation and measures derived from the VGR task as well as scores from the FMA-UE and CMSA. Impairments in adaptation were statistically independent from impairments in movement, assessed on the VGR task, FMA-UE, and CMSA in our sample of participants with stroke.

### 4.1. Visually-Guided Reaching, FMA Scores, and CMSA Scores Account for Relatively Little Variance in Visuomotor Adaptation

We observed a range of adaptation and visually-guided reaching abilities in our sample of participants with stroke. We quantified the relationships between individual measures of adaptation and upper-limb movements, assessed robotically and with traditional clinical scales including the FMA-UE and CMSA. The robotic assessment allowed for us to understand how adaptation relates to overall reaching performance as well as specific aspects of movement. In general, the relationships between measures of adaptation and visually-guided reaching were of weak-to-moderate strength, accounting for, at most, a third of the ranked variance in measures of adaptation (rho^2^ ≤ 34.0%). Scores in the FMA-UE and CMSA also accounted for very little of the ranked variance in measures of adaptation (rho^2^ ≤ 13.4%). Thus, assessing an individual’s ability to perform upper-limb movements, whether robotically or via common clinical scales, may provide little information about the ability to adapt their reaching movements with practice.

What other factors are associated with adaptation after stroke? Previous studies have shown that the side of the more affected limb may influence adaptation after stroke [12,13,17,66]. We repeated our analyses accounting for the side of the more affected limb in right-handed participants (dominant or non-dominant), owing to a limited sample of participants who were left-handed prior to stroke (*n* = 2, see limitations for more details). The analysis again revealed weak-to-moderate correlations between adaptation and reaching, with impairments in adaptation being independent from impairments in visually-guided reaching. The consistency of our findings in right-handed individuals (*n* = 39) helps mitigate the concern that our results are confounded by differences in limb dominance prior to stroke or the effects of the arm more affected by stroke. Other research has shown that individuals with visual impairments that affect their ability to perform daily activities can have impairments in visuomotor adaptation [67]. Visual impairments are complex, and further studies employing detailed assessments of oculomotor control or visual field deficits are needed to understand if these impairments interact with the ability to use visual feedback to adapt arm movements after stroke.

In healthy adults, factors like spatial working memory [68,69], cognitive strategies [70], and proprioception may be important for visuomotor adaptation [71,72]. In contrast, recent studies have shown that cognitive impairments [73] as well as impairments in position- and kinesthetic-sense [16] may not be closely related to visuomotor adaptation after stroke. Thus, there are discrepancies between research in healthy adults and individuals with stroke, such that knowing the relationship between proprioception, cognitive strategies, and motor learning may not be informative when stroke damages the brain and causes heterogenous and highly individualized impairments. This stresses the need for additional studies that examine how the heterogeneity of impairments (e.g., visual, proprioception) and the location and size of stroke lesions impact visuomotor adaptation and other forms of motor learning. The approach would help to characterize the range of distinct impairments that can present after stroke and provide insight into how stroke impacts basic motor learning mechanisms.

The relationship between adaptation and movement variability is a subject of debate. Some evidence suggests that increased movement variability might reflect exploratory behaviors that enable faster adaptation to reinforcement [74,75]. In error-based adaptation, some studies in healthy adults suggest that greater movement variability is associated with reduced adaptation rates [74], although others have reported positive, negative, and null associations that may depend on features of the task [75]. We found that *Trials to Adapt* correlated with *VGR Task Score*, *IDE*, and *MT* in the stroke group. Moreover, *Trials to Adapt* but not *Initial* or *Final Adaptation* correlated positively with movement variability in participants with stroke, emphasizing that individuals with more variable movements may require more *Trials to Adapt* (Supplementary Materials S3). The *Trials to Adapt* measure captures the number of trials taken to adapt consistently above the 5% bound of *Final Adaptation* in the control sample. Thus, *Trials to Adapt* reflects not only how quickly a participant adapts to normal levels but also the consistency with which they can adapt their movements. In the VGR task, more variable initial movements can result in a greater *IDE* value [25]. Initiating movement in the wrong direction also requires a correction to arrive in the end-target, which may result in greater *MT* and *VGR Task Scores* [25]. Collectively, our results suggest that individuals with more variable reaching patterns may require more *Trials to Adapt*, although it is likely that other factors also influence adaptation following stroke.

### 4.2. Reaching Impairments Do Not Preclude Normal Visuomotor Adaptation

We used Fisher’s exact test to assess categorical relationships between distinct phenotypes of impairment across the visuomotor adaptation and visually-guided reaching tasks as well as clinical assessments of sensory and motor function. The results showed that impairments in adaptation were statistically independent from movement impairments assessed using robotics and traditional clinical assessments like the FMA and CMSA. Naturally, this independence was related to the variety of unique impairment profiles expressed in our stroke sample. Some individuals had impairments in visuomotor adaptation and reaching (Figure 2C). One participant had impaired adaptation and normal reaching, while a notable proportion (range [36.6%, −65.9%]) of our sample had normal adaptation and reaching impairments (Figure 2B). This means that, among those with VGR impairments, the distribution of (non-)impairments in adaptation was random. The sub-group of participants with normal adaptation and a range of reaching impairments (*VGR Task Score*: range [2.24, −7.11]) is of particular interest because it suggests that movement impairments may not always be a good indicator of an individual’s capacity to adapt their movements with practice. It is possible that this sub-group represents individuals that may benefit from motor learning-based interventions and rehabilitation strategies (e.g., robotic rehabilitation). In contrast, individuals with impairments in adaptation and visually-guided reaching may benefit from therapy that attempts to augment and restore the capacity for motor control and learning [18,19,20]. Likewise, individuals with normal movements may have motor learning impairments that benefit from therapy aimed at improving motor learning ability. This could be important because, despite having normal movements, motor learning impairments could influence how individuals learn new tasks or adapt learned movements that are important for their occupation or recreational activities. Having assessment tools to identify whether a patient has motor learning impairments may help researchers understand whether they are related to the long-term recovery of movements after stroke [4,76]. It is possible that several assessments are needed to capture impairments in different facets of motor learning [4,74]. The integration of these assessments into clinical research and practice could be an important step, however, in developing new, personalized strategies to rehabilitate individuals with motor learning impairments.

### 4.3. Performance Versus Impairment

We used robotics to characterize the relationship between adaptation and visually-guided reaching performance. We also used normative datasets to identify and test the independence of impairments in adaptation and reaching. The two statistical approaches each serve a distinct purpose. The correlation analysis provides insight into the strength and directionality of associations between adaptation and performance in the visually-guided reaching task or clinical assessments of upper-limb movements. Examining broad ranges of values can deepen our understanding of motor learning from a research perspective. However, this approach may be less practical in a clinical setting where resources are limited, and therapists must distinguish between normal and impaired behavior for the purpose of planning therapy. Impairment is categorical and refers to a disruption of a specific process relative to normal performance in control participants. To illustrate this point, consider a 68-year-old, left-handed male with an average initial direction error (*IDE*) of 6.30° when performing reaching movements with his more affected left arm. Should their rehabilitation focus on reducing these directional errors? This question is difficult to answer without knowing the range of directional errors that is considered normal while accounting for the participant’s age, sex, and handedness. Understanding how impairments in adaptation and visually-guided reaching relate could also be valuable when planning therapy. For example, if this individual has impairments in adaptation and reaching, will working on one impairment help with addressing the other? The results from our sample suggest that impairments in adaptation and reaching may be independent, such that individuals with both impairments may benefit from the integration of therapy that focuses on improving motor learning and motor function.

### 4.4. Limitations and Future Directions

This study examined a specific aspect of motor learning that is referred to as visuomotor adaptation [4]. It should be noted that adapting reaching movements to a systematic visual disturbance in a laboratory setting may seem far removed from the tasks performed in a rehabilitation clinic. However, visuomotor adaptation can serve as a useful model to understand motor learning after stroke by providing insight into the ways that patients use visual feedback to adapt their movements [17]. More research is needed to understand how impairments in visuomotor adaptation relate to other types of motor learning after stroke, such as the ability to adapt to novel forces that disrupt the motion of the arm, learn new sequences of arm movements, or other motor skills [4,76].

We recruited individuals with the capacity to understand task instructions and perform repeated movements in a robotic exoskeleton. Individuals with little to no movement in their stroke-affected limb would have been unable to perform the task. Consequently, our sample consisted of individuals with mild-to-moderate movement impairments, which limits our ability to comment on visuomotor adaptation amongst participants with more severe impairments. This may have also led us to underestimate the prevalence of impairments in adaptation and visually-guided reaching. Our sample of left-handed individuals was small, and although consistent with population statistics, this made it difficult to address questions surrounding the laterality of stroke lesions (left or right hemisphere) and side of the more affected arm (dominant vs. non-dominant). Larger studies are needed to fully disentangle the roles of laterality and dominance on visuomotor adaptation.

The creation of large normative datasets to characterize normal and impaired behavior is a challenge faced when developing robotic assessments. Developing these datasets is a time- and resource-intensive process. The benefit is that they can be used to identify patient-specific impairments in view of what is normal for the participant’s age, sex, and handedness. Although the normative dataset for the VGR task has been in collection for many years [25], we have only recently started to build a normative dataset for the VMR task. The normative dataset for the VMR task was small in our study, meaning that we were not able to calculate a *Task Score* like in the VGR task.

The VMR and VGR tasks differed in design. The VMR task involved reaching to a single target, while the VGR task involved reaching from a central target to one of eight peripheral targets. Comparing performance across motor tasks using correlational and independence analyses is common in movement neuroscience [74,77,78,79]. While some tasks show low inter-task correlations, highlighting task-specific processes, others, especially reaching tasks, often reveal overlapping outcomes despite differences in task design [77,80]. Correlation analyses remain essential for validating novel assessments, understanding shared or dissociable sensorimotor functions, and interpreting individualized patterns of motor impairment [81,82]. For example, reaching and proprioceptive tasks are behaviorally and neurally dissociable post-stroke [5,9,16,21,28,38,41,83,84,85]. A recent study [80] pooled data from four reaching tasks performed by healthy participants (*n* = 2185). The tasks used different movement directions (1–8 targets) and apparatuses (robotic manipulandum, stylus, trackpad, joystick), yet found consistent effects of age and sex on movement outcomes, suggesting that diverse reaching tasks can capture common aspects of motor performance.

Nevertheless, some studies suggest the neural control of movements may differ between single- and multiple-target tasks [86,87,88]. This may have led to more consistent reaching patterns in the single-target VMR task compared to the VGR task. We repeated our analysis, however, by calculating the IDE measure used in visually-guided reaching derived from the baseline phase of the VMR task. The approach allowed for us to examine the relationship between reaching and adaptation in a single-target task. The supplemental analyses were qualitatively consistent with the results from the normative VGR dataset, highlighting the robustness of our findings both within (single-target VMR task) and between tasks (single-target VMR vs. multitarget VGR task). Collectively, the results suggest that the weak–moderate relationships in performance and independence of impairments in the VMR and VGR tasks do not solely reflect differences in task design (Supplementary Materials S3).

Finally, our study was cross-sectional and is limited in its ability to comment on the relationship between visuomotor adaptation and the recovery of movements after stroke. There is a need for longitudinal studies that track impairments in motor adaptation over the time course of stroke recovery. Such studies can provide more insight into the ways in which motor adaptation and its relationship with rehabilitation outcomes evolve over the course of stroke recovery.

## 5. Conclusions

Impairments in visuomotor adaptation are relatively common after stroke (46.3–53.0%) [16,17]. Here, we show that the relationships between adaptation and visually-guided reaching and clinical assessments of arm function were weak to moderate, at best. Moreover, impairments in visuomotor adaptation and movement, assessed clinically and robotically, were statistically independent (i.e., randomly distributed), highlighting that these forms of impairment were not closely related in our sample. These findings suggest that assessing motor learning, in addition to motor function, may be important after stroke and raises questions about other factors that may influence and interact with visuomotor adaptation.

## Figures and Tables

**Table 1:** Demographics and clinical characteristics of participants with stroke.

Demographics	Control	Stroke
N	41	41
Age	62 [33–77]	65 [27–88]
Sex (F:M)	22:19	17:24
Handedness (L:R)	6:35	2:39
**Clinical Measures**		
Affected Arm (Dominant:Non-dominant)		26:15
Stroke Type (Ischemic:Hemorrhagic)		36:5
Days from Stroke to Robotic Assessment		31 [3–1102]
Lesion Volume (mL)		8.72 [0.25–97.10]
FMA (/66) ƗƗƗ		62 [35–66]
CMSA Arm (/7) Ɨ		6 [3–7]
PPT ƗƗ		6 [0–15]
MRC Arm Strength composite (/45) Ɨ		43 [20–45]
MAS (/4) Ɨ		0 [0–2]
TLT (/3) Ɨ		0 [0–3]
FIM (/126) ƗƗƗƗ		115 [87–126]

Demographic and clinical measures are presented as median [range]. Fugl–Meyer Assessment of Motor Recovery–Upper Extremity (FMA; normal = 66), Chedoke–McMaster Stroke Assessment–Arm (CMSA; normal = 7), Purdue Peg Test (PPT), Medical Research Council Strength Assessment–Arm Strength (MRC; normal = 45), Modified Ashworth Scale (MAS; normal = 0; scale = 0, 1, 1+, 2, 3, 4), Thumb Localization Test (TLT; normal = 0), and Functional Independence Measure (FIM; normal = 126). ^Ɨ^ CMSA, MRC, TLT, and MAS scores were obtained for 36 participants. ^ƗƗ^ PPT was assessed in 33 participants. ^ƗƗƗ^ FMA was obtained for 35 participants. ^ƗƗƗƗ^ FIM was obtained for 37 participants.

## Data Availability

The data used in the current study are not publicly available. Data may be made available from the corresponding author upon reasonable request.

## Supplementary Materials

The following supporting information can be downloaded at: https://www.mdpi.com/article/10.3390/brainsci15060619/s1, Table S1: Description of VGR Task Measures. Figure S1: Scatterplots of VGR Task Score and (A) Initial Adaptation, (B) Final Adaptation, and (C) Trials to Adapt for participants with stroke. Scatterplots have been divided into 4 quadrants: participants with normal reaching and adaptation (open diamond), participants with impaired adaptation (solid diamond), participants with impaired reaching (solid circle), and participants with impaired reaching and adaptation (open circle). Spearman’s correlations indicate the strength of ranked associations. Barnard’s tests assessed for categorical relationships between impairments. * indicates *p* < 0.05. (D) Spearman’s correlations and Barnard’s tests of independence for individual measures derived from the VGR and VMR tasks. Upper right: Spearman’s rho values are presented, and darker boxes indicate stronger correlations. Bold white numbers indicate significant correlations between variables (*p* < 0.05 after Bonferroni–Holm corrections—21 correlations). Bottom left: Wald’s statistic from the Barnard’s tests of independence are presented, and black boxes with bold white numbers indicate significant categorical associations between variables (*p* < 0.05 after Bonferroni–Holm corrections—21 tests). Table S2: Adaptation vs Reaching Variables Derived From the Baseline Phase of the VMR Task – Spearman’s Rho and Fisher’ Exact Test. Table S3: Adaptation vs Reaching Variables Derived From the Baseline Phase of the VMR Task – Barnard’s Test. Table S4: VMR vs VGR Score with Dominant Arm Impaired as a Covariate. Table S5: VMR vs Individual VGR Variables with Dominant Arm Impaired as a Covariate. Table S6: VMR vs VGR Score Only in R-Handed Participants. Table S7: VMR vs Individual VGR Variables Only in R-Handed Participants

## Abbreviations

**ADC**	Apparent Diffusion Coefficient
**CMSA**	Chedoke–McMaster Stroke Assessment—Arm Impairment Inventory
**CT**	Computed Tomography
**DWI**	Diffusion Weighted Imaging
**FIM**	Functional Independence Measure
**FLAIR**	T2 Fluid-Attenuated Inversion Recovery
**FMA**	Fugl–Meyer Assessment of Motor Recovery—Upper Extremity Motor Assessment
**GRE**	Gradient Echo
**IDE**	Initial Direction Error
**MAS**	Modified Ashworth Scale
**MNI**	Montreal Neurological Institute
**MRC**	Medical Research Council Strength Score Composite
**MRI**	Magnetic Resonance Imaging
**MT**	Movement Time
**PLR**	Path Length Ratio
**RSS**	Root Sum Square
**SMC**	Speed Maxima Count
**SRRR**	Stroke Recovery and Rehabilitation Roundtable
**SWI**	Susceptibility Weighted Imaging
**TLT**	Thumb Localization Test
**VGR**	Visually-Guided Reaching
**VMR**	Visuomotor Rotation
