# Supplementary material for: Movement Impairments May Not Preclude Visuomotor Adaptation After Stroke
Source: Brain Sci. 2025 Jun 8;15(6):619. doi: 10.3390/brainsci15060619 (PMC12191063; doi:10.3390/brainsci15060619)
Supplement: Supplementary file 1 [file brainsci-15-00619-s001.zip › Supplementary Materials S1 - VGR Variables 20250607.pdf]

Table S1: Description of VGR Task Measures

VGR Task Measure	Description
**No Initial Stabilization**	Number of trials in which the participant did not stabilize at the start target.
**No End Movement**	Number of trials in which movement offset was not detected.
**Posture Speed**	The participant's median hand speed when holding the cursor in the start target.
**Reaction Time**	The elapsed time between the illumination of the peripheral end-target and movement onset.
**Initial Direction Error**	The unsigned angular deviation of the hand path relative to a straight line between the start and end targets at the completion of the initial movement.
**Initial Distance Ratio**	The ratio of the distance travelled in the first sub-movement relative to the entire movement distance.
**Speed Maxima Count**	The number of speed maxima that occur between movement onset and offset.
**Min-Max Speed**	The mean difference between local hand speed minima and maxima for all pairs found between movement onset and offset.
**Movement Time**	The elapsed time between movement onset and offset.
**Path Length Ratio**	The ratio between the distance travelled by the hand and distance between the start and end target.
**Max Speed**	The global maximum in hand speed between movement onset and offset.
