# Supplementary material for: Movement Impairments May Not Preclude Visuomotor Adaptation After Stroke
Source: Brain Sci. 2025 Jun 8;15(6):619. doi: 10.3390/brainsci15060619 (PMC12191063; doi:10.3390/brainsci15060619)
Supplement: Supplementary file 1 [file brainsci-15-00619-s001.zip › Supplementary Materials S2 - Main Results Using Barnard's Test 20250607.pdf]

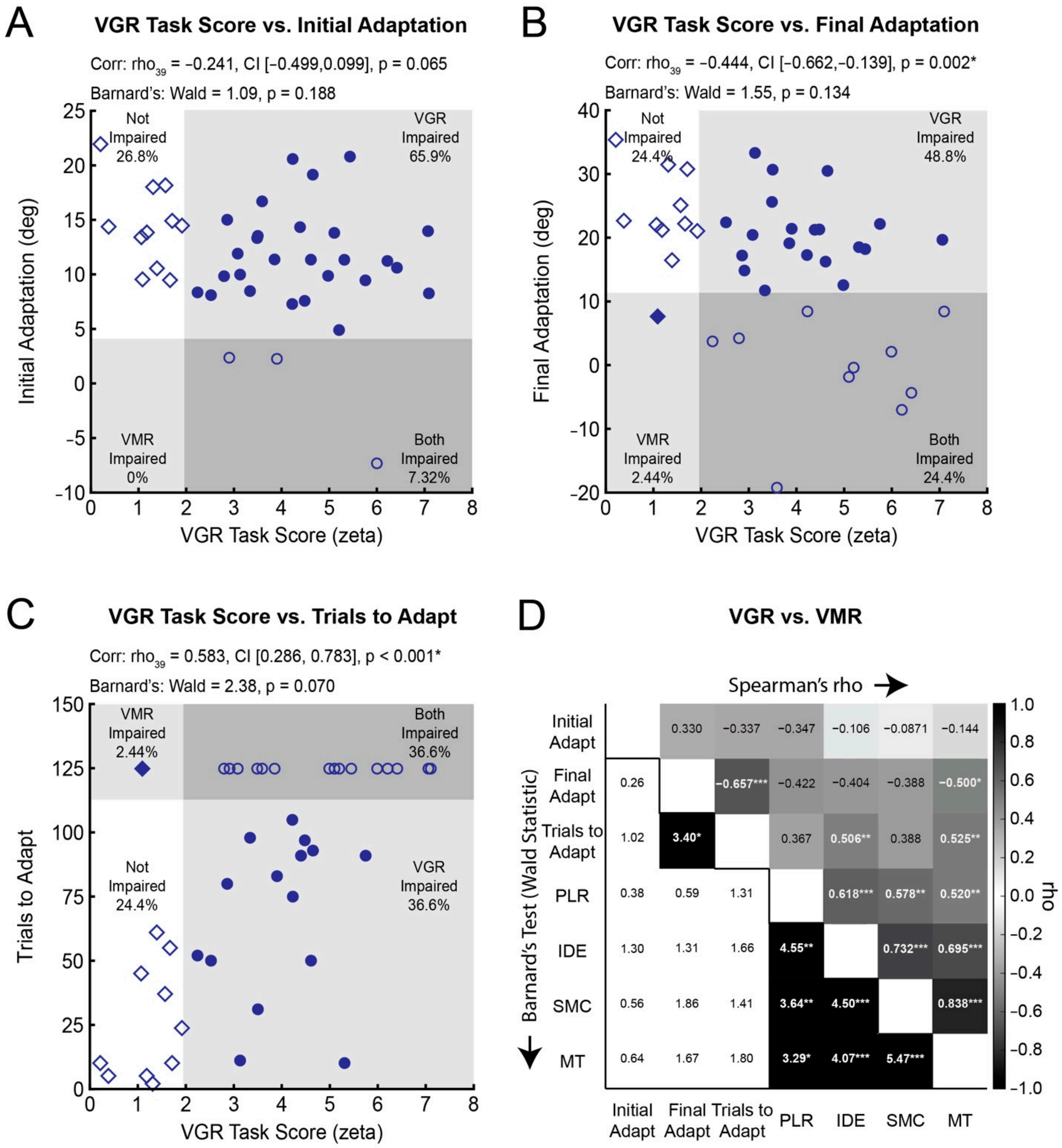
A
VGR Task Score vs. Initial Adaptation
Corr: rho39 = −0.241, CI [−0.499,0.099], p = 0.065
Barnard's: Wald = 1.09, p = 0.188
Not Impaired 26.8%
VGR Impaired 65.9%
VMR Impaired 0%
Both Impaired 7.32%
Initial Adaptation (deg)
VGR Task Score (zeta)
B
VGR Task Score vs. Final Adaptation
Corr: rho39 = −0.444, CI [−0.662,−0.139], p = 0.002*
Barnard's: Wald = 1.55, p = 0.134
Not Impaired 24.4%
VGR Impaired 48.8%
VMR Impaired 2.44%
Both Impaired 24.4%
Final Adaptation (deg)
VGR Task Score (zeta)
C
VGR Task Score vs. Trials to Adapt
Corr: rho39 = 0.583, CI [0.286, 0.783], p < 0.001*
Barnard's: Wald = 2.38, p = 0.070
VMR Impaired 2.44%
Both Impaired 36.6%
Not Impaired 24.4%
VGR Impaired 36.6%
Trials to Adapt
VGR Task Score (zeta)
D
VGR vs. VMR
Spearman's rho
Barnard's Test (Wald Statistic)
Initial Adapt
Final Adapt
Trials to Adapt
PLR
IDE
SMC
MT
0.330 −0.337 −0.347 −0.106 −0.0871 −0.144
0.26 −0.657*** −0.422 −0.404 −0.388 −0.500*
1.02 3.40* 0.367 0.506** 0.388 0.525**
0.38 0.59 1.31 0.618*** 0.578** 0.520**
1.30 1.31 1.66 4.55** 0.732*** 0.695***
0.56 1.86 1.41 3.64** 4.50*** 0.838***
0.64 1.67 1.80 3.29* 4.07*** 5.47***
rho
