# Supplementary material for: Movement Impairments May Not Preclude Visuomotor Adaptation After Stroke
Source: Brain Sci. 2025 Jun 8;15(6):619. doi: 10.3390/brainsci15060619 (PMC12191063; doi:10.3390/brainsci15060619)
Supplement: Supplementary file 1 [file brainsci-15-00619-s001.zip › Supplementary Materials S3 - VGR Derived From Baseline and Side Affected by Stroke 20250607.pdf]

Table S2: Adaptation vs Reaching Variables Derived From the Baseline Phase of the VMR Task – Spearman's Rho and Fisher' Exact Test

**N = 41**	**Initial Adaptation**	**Final Adaptation**	**Trials to Adapt**	**PLR**	**$IDE_{SpeedMin}$**	**IDE_{150ms}**	**SMC**	**MT**	**BVar**
**Initial Adapt**		rho = 0.330 (*p* = 0.493)	rho = -0.337 (*p* = 0.465)	rho = -0.189 (*p* = 0.238)	rho = -0.145 (*p* = 0.365)	rho = -0.007 (*p* = 0.966)	rho = -0.014 (*p* = 0.931)	rho = -0.186 (*p* = 0.246)	rho = -0.056 (*p* = 0.729)
**Final Adapt**	OR = 1.40, (*p* = 1.00)		**rho = -0.657 (*p* < 0.001)***	rho = -0.212 (*p* = 0.184)	rho = -0.239 (*p* = 0.133)	rho = -0.005 (*p* = 0.974)	rho = -0.356 (*p* = 0.358)	rho = -0.457 (*p* = 0.056)	rho = -0.114 (*p* = 0.479)
**Trials to Adapt**	OR = 3.43, (*p* = 0.550)	**OR = 14.8, (*p* = 0.040)***		rho = 0.429 (*p* = 0.098)	**rho = 0.548 (*p* = 0.006)****	rho = 0.385 (*p* = 0.222)	**rho = 0.524 (*p* = 0.012)***	**rho = 0.553 (*p* = 0.005)****	**rho = 0.493 (*p* = 0.027)***
**PLR**	OR = ∞, (*p* = 0.091)	OR = 1.57, (*p* = 0.725)	OR = 2.96, (*p* = 0.120)		**rho = 0.706 (*p* < 0.001)*****	rho = 0.277 (*p* = 0.080)	rho = 0.218 (*p* = 0.172)	rho = 0.068 (*p* = 0.674)	**rho = 0.498 (*p* = 0.024)***
**$IDE_{SpeedMin}$**	OR = ∞, (*p* = 0.539)	OR = 0.875, (*p* = 1.00)	OR = 3.40, (*p* = 0.176)	**OR = 26.0, (*p* = 0.012)***		**rho = 0.486 (*p* = 0.029)***	**rho = 0.573 (*p* = 0.003)****	**rho = 0.479 (*p* = 0.033)***	**rho = 0.684 (*p* < 0.001)*****
**IDE_{150ms}**	OR = 0, (*p* = 1.00)	OR = 0.650, (*p* = 1.00)	OR = 8.00, (*p* = 0.067)	OR = 0.745, (*p* = 1.00)	OR = ∞, (*p* = 0.137)		rho = 0.435 (*p* = 0.089)	rho = 0.408 (*p* = 0.145)	**rho = 0.809 (*p* < 0.001)*****
**SMC**	OR = 0, (*p* = 1.00)	OR = 0, (*p* = 1.00)	OR = ∞, (*p* = 0.390)	OR = ∞, (*p* = 0.463)	OR = ∞, (*p* = 0.067)	OR = 0, (*p* = 1.00)		**rho = 0.861 (*p* < 0.001)*****	**rho = 0.600 (*p* = 0.001)*****
**MT**	OR = 0, (*p* = 1.00)	OR = ∞, (*p* = 0.268)	OR = ∞, (*p* = 0.390)	OR = 0, (*p* = 1.00)	OR = ∞, (*p* = 1.00)	OR = 0, (*p* = 1.00)	OR = 0, (*p* = 1.00)		**rho = 0.491 (*p* = 0.027)***
**BVar**	OR = 7.50, (*p* = 0.142)	OR = 1.23, (*p* = 1.00)	OR = 3.15, (*p* = 0.150)	OR = 18.9, (*p* = 0.084)	OR = ∞, (*p* = 0.281)	OR = (6.21, *p* = 0.084)	OR = 0, (*p* = 1.00)	OR = 0, (*p* = 1.00)	

Note: p-values are Bonferonni-Holm corrected. All variables were derived from the VMR task. PLR, $IDE_{SpeedMin}$, IDE_{150ms}, SMC, MT, BVar were measured over the 25 trials of the baseline phase.

Table S3: Adaptation vs Reaching Variables Derived From the Baseline Phase of the VMR Task – Barnard's Test

N = 41	Initial Adaptation	Final Adaptation	Trials to Adapt	PLR	$IDE_{SpeedMin}$	IDE_{150ms}	SMC	MT	BVar
**Initial Adapt**									
**Final Adapt**	Wald = 0.264, (*p* =0.753)								
**Trials to Adapt**	Wald =1.02, (*p* = 0.258)	**Wald = 3.40, (*p* = 0.033)***							
**PLR**	Wald = 1.94, (*p* = 1.00)	Wald = 0.638, (*p* = 0.294)	Wald = 0.166, (*p* = 0.058)						
**$IDE_{SpeedMin}$**	Wald = 1.30, (*p* = 0.155)	Wald = 0.181, (*p* = 0.467)	Wald = 1.66, (*p* = 0.058)	**Wald = 3.62, (*p* = 0.007)****					
**IDE_{150ms}**	Wald = 0.671, (*p* = 0.414)	Wald = 0.368, (*p* = 0.452)	Wald = 2.00, (*p* = 0.954)	Wald = 0.304, (*p* = 0.655)	Wald = 1.83, (*p* = 0.061)				
**SMC**	Wald = 0.285, (*p* = 0.660)	Wald = 0.613, (*p* = 0.294)	Wald = 1.27, (*p* = 0.184)	Wald = 1.09, (*p* = 0.328)	Wald = 3.87, (*p* = 0.811)	Wald = 0.377, (*p* = 0.747)			
**MT**	Wald = 0.285, (*p* = 0.660)	Wald = 1.67, (*p* = 0.116)	Wald = 1.27, (*p* = 0.184)	Wald = 0.941, (*p* = 0.393)	Wald = 0.729, (*p* = 0.509)	Wald = 0.377, (*p* = 0.747)	Wald = 0.160, (*p* = 0.889)		
**BVar**	Wald = 1.77, (*p* = 0.117)	Wald = 0.260, (*p* = 0.482)	Wald = 1.56, (*p* = 0.118)	Wald = 3.18, (*p* = 0.029)	Wald = 2.62, (*p* = 0.232)	Wald = 1.98, (*p* = 0.053)	Wald = 0.575, (*p* = 0.608)	Wald = 0.575, (*p* = 0.608)	

Note: p-values are Bonferonni-Holm corrected. All variables were derived from the VMR task. PLR, $IDE_{SpeedMin}$, IDE_{150ms}, SMC, MT, BVar were measured over the 25 trials of the baseline phase.

Table S4: VMR vs VGR Score with Dominant Arm Impaired as a Covariate

N = 41	Initial Adaptation	Final Adaptation	Trials to Adapt
**VGR Score (partial correlation)**	rho_p = -0.254 (*p* = 0.114)	**rho_p = -0.415 (*p* = 0.016)***	**rho_p = 0.584 (*p* < 0.001)*****
**VGR Score (Log Regress model p-value)**	*p* = 0.375	*p* = 0.054	*p* = 0.071

Note: p-values are Bonferonni-Holm corrected.

Table S5: VMR vs Individual VGR Variables with Dominant Arm Impaired as a Covariate

N = 41	Initial Adaptation	Final Adaptation	Trials to Adapt	PLR	IDE	SMC	MT
**Initial Adapt**		rho$_p$ = 0.356 (*p* = 0.160)	rho$_p$ = -0.343 (*p* = 0.145)	rho$_p$ = -0.346 (*p* = 0.145)	rho$_p$ = -0.116 (*p* = 0.478)	rho$_p$ = -0.096 (*p* = 0.556)	rho$_p$ = -0.168 (*p* = 0.301)
**Final Adapt**	*p* = 0.967		**rho$_p$ = -0.660 (*p* < 0.001)*** **	**rho$_p$ = -0.455 (*p* = 0.038)***	rho$_p$ = -0.373 (*p* = 0.157)	rho$_p$ = -0.359 (*p* = 0.160)	**rho$_p$ = -0.450 (*p* = 0.039)***
**Trials to Adapt**	*p* = 0.603	***p* = 0.002****		rho$_p$ = 0.374 (*p* = 0.159)	**rho$_p$ = 0.499 (*p* = 0.014)***	rho$_p$ = 0.379 (*p* = 0.159)	**rho$_p$ = 0.525 (*p* = 0.007)****
**PLR**	*p* = 0.922	*p* = 0.051	*p* = 0.366		**rho$_p$ = 0.639 (*p* < 0.001)*****	**rho$_p$ = 0.597 (*p* < 0.001)*****	**rho$_p$ = 0.570 (*p* = 0.002)****
**IDE**	*p* = 0.266	*p* = 0.428	*p* = 0.227	***p* < 0.001*****		**rho$_p$ = 0.724 (*p* < 0.001)*****	**rho$_p$ = 0.684 (*p* < 0.001)*****
**SMC**	*p* = 0.826	*p* = 0.279	*p* = 0.358	***p* = 0.003****	***p* < 0.001*****		**rho$_p$ = 0.840 (*p* < 0.001)*****
**MT**	*p* = 0.773	*p* = 0.374	*p* = 0.192	***p* = 0.009****	***p* < 0.001*****	***p* < 0.001*****	

Note: p-values are Bonferonni-Holm corrected.

Table S6: VMR vs VGR Score Only in R-Handed Participants

N = 39	Initial Adaptation	Final Adaptation	Trials to Adapt
**VGR Score (correlation)**	rho$_p$ = -0.25 (*p* = 0.062)	**rho$_p$ = -0.457 (*p* = 0.004)****	**rho$_p$ = 0.555 (*p* < 0.001)*****
**VGR Score (Fisher's Exact Test)**	OR = ∞, (p = 1.00)	OR = 5.56, (p = 0.130)	**OR = 0.083, (p = 0.041)***

Note: p-values are Bonferonni-Holm corrected.

Table S7: VMR vs Individual VGR Variables Only in R-Handed Participants

N = 39	Initial Adaptation	Final Adaptation	Trials to Adapt	PLR	IDE	SMC	MT
**Initial Adapt**		rho_p = 0.401 (*p* = 0.076)	rho_p = -0.362 (*p* = 0.094)	rho_p = -0.415 (*p* = 0.082)	rho_p = -0.101 (*p* = 0.859)	rho_p = -0.130 (*p* = 0.859)	rho_p = -0.190 (*p* = 0.743)
**Final Adapt**	OR = 2.70, (p = 0.4899)		**rho_p = -0.688 (*p* < 0.001)*****	**rho_p = -0.450 (*p* = 0.045)***	rho_p = -0.417 (*p* = 0.082)	rho_p = -0.410 (*p* = 0.078)	**rho_p = -0.466 (*p* = 0.036)***
**Trials to Adapt**	OR = 1.64, (p = 1.00)	**OR = 16.5, (p 0.013)***		rho_p = 0.400 (*p* = 0.080)	**rho_p = 0.463 (*p* = 0.036)***	rho_p = 0.395 (*p* = 0.070)	**rho_p = 0.500 (*p* = 0.017)***
**PLR**	OR = ∞, (p = 0.5024)	OR = 2.33, (p = 0.3092)	OR = 1.71, (p = 0.5194)		**rho_p = 0.625 (*p* < 0.001)*****	**rho_p = 0.576 (*p* = 0.002)****	**rho_p = 0.572 (*p* = 0.002)****
**IDE**	OR = ∞, (p = 0.5277)	OR = 3.94, (p = 0.1519)	OR = 4.00, (p = 0.0965)	**OR = 172.5, (p < 0.001)*****		**rho_p = 0.737 (*p* < 0.001)*****	**rho_p = 0.731 (*p* < 0.001)*****
**SMC**	OR = 1.29, (p = 1.00)	OR = 5.33, (p = 0.500)	OR = 4.50, (p = 0.632)	**OR = 15.0, (p = 0.009)****	**OR = ∞, (p < 0.001)*****		**rho_p = 0.881 (*p* < 0.001)*****
**MT**	OR = 0.950, (p = 1.00)	OR = 3.49, (p = 0.1589)	OR = 4.40, (p = 0.0516)	**OR = 14.0, (p = 0.007)****	**OR = 60.0, (p < 0.001)*****	**OR = ∞, (p = < 0.001)*****	

Note: p-values are Bonferonni-Holm corrected.
